
JOURNAL INFORMATION
==============================
NLM Title Abbreviation: J Child Orthop
Journal ID: J Child Orthop
NLM Journal ID: 101313582

Journal of Children's Orthopaedics

ISSN: 1863-2521
EISSN: 1863-2548
Publisher: SAGE Publications

ARTICLE INFORMATION
==============================
PMCID: PMC4353817
DOI: 10.1007/s11832-015-0645-0
Article ID: 645
Article version: 1
Subjects: Author Index

Author Index IFPOS, 2015, France

Electronic publication date: 2015 Feb 25
Print publication date: 2015 Apr
Volume: 9
Issue: Suppl 1
First page: 9
Last page: 10
Copyright: © The Author(s) 2015
License: This article is distributed under the terms of the Creative Commons Attribution License which permits any use, distribution, and reproduction in any medium, provided the original author(s) and the source are credited.
License URL: https://creativecommons.org/licenses/by/4.0/

issue-copyright-statement: © The Author(s) 2015

==============================

Abdel-Ghani, Hisham IEP8
Abousamra, Oussama IOP1
Accadbled, Franck IOP18
Ahn, Yeong Seub IEP10
Ahn, Tae Young IOP22
Allet, Lara IEP6
Amy, Zavatsky IOP28
Armand, Stephane IOP6
Armand, Stéphane IEP6

Bayhan, Ilhan A IOP3 IOP1
Biassoni, Lorenzo IOP27
Blasier, Dale IOP23
Bonnefoy-mazure, Alice IOP6
Bonnefoy-Mazure, Alice IEP6

Campos, Felipe IOP5
Cavalheiro, Camila IOP5
Chang, Wei-Ning IOP26
Chang, Chia-Hsieh IOP8
Chang, Chia IEP2
Chang, Liang IEP2
Chang, Chia IEP3 IOP10
Chen, Hsuan-Yu IEP4 IOP12
Chen, Joi IOP23
Cheng, Pen-Gang IOP26
Cho, Tae-joon IEP11
Choi, In Ho IEP11
Chomiak, Jirí IEP12
Chomiak, Jiri IEP14
Couto, Juan IOP20
Cowling, Paul IEP7
Craig, Joshua IEP7

Dabney, Kirk W IOP1
de Coulon, Geraldo IOP6
de Coulon, Geraldo IEP6
Dungl, Pavel IEP12 IEP14

Eastwood, Deborah IOP27
Easty, Marina IOP27
Eckhardt, Adam IEP14
Endo, Naoto IOP7

Frydrychová, Monika IEP12 IEP14
Fucs, Patricia IOP5 IEP5

Galal, Sherif IEP1 IOP16
Gegenleitner, Kathrin IOP4
Glorion, Christophe IOP19

Hailer, Yasmin D. IOP14
Hosny, Gamal IEP9
Hwang, Jin Ho IEP13

Ibnoulkhatib, Aissa IOP18

Jung, Sung Taek IEP10 IOP13

Kakizaki, Jun IOP7
Kam, Minjae IOP25
Kamegaya, Makoto IOP7
Kang, Seungcheol IOP25
Kao, Hsuan-Kai IOP8 IEP3 IOP10
Khouri, Nejib IOP19
Kim, Han-Ju IOP11
Kim, Hui Taek IOP15 IOP22
Kothari, Alpesh IOP28
Kraus, Tanja IOP4
Kuo, Ken IEP3 IOP10
Kuo, Ken N. IEP4 IOP12

Lascombes, Pierre IOP6
Lascombes, Oierre IEP6
Lee, Wei-Chun IOP8
Lee, Soon Hyuck IOP17 IOP9
Lee, Wei-Chun IEP3 IOP10
Lee, Wei Chun IOP2
Lee, Ji-Ho IOP11
Lee, Tae Hoon IOP15
Lim, Kuk Pil IOP22

MA, Ruixue IOP24
Mahmoud, Mostafa IEP8
Maisongrosse, Paul IOP18
Miladi, Lotfi IOP19
Miller, Freeman IOP3 IOP1
Miraj, Faisal IOP25
Montgomery, Richard IEP7
Murakami, Reiko IOP7

Na, Bo Ram IOP13
Nishnianidze, Tristan IOP3 IOP1

Odent, Thierry IOP19
Oh, Chang Seon IEP10 IOP13
Ostadal, Martin IEP14
Oštádal, Martin IEP12

Pailhes, Régis IOP18
Park, Kwang-Won IOP11
Park, Soo-Sung IOP25
Plas, Benjamin IOP18

Rahbek, Ole IOP27
Rangan, Amar IEP7
Rogers, Kenneth J IOP3 IOP1

Sagawa, Yoshi IEP6
Sagawa Jr, Yoshi IOP6
Saisu, Takashi IOP7
Sales de gauzy, jerome IOP18
Sees, Julie IOP3
Shen, Shih-Hsun IEP3
Simionato, Marcos IOP5
Singer, Georg IOP4
Song, Hae-Ryong IOP11
Song, Mi Hyun IEP11
Stebbins, Julie IOP28
Steinwender, Gerhardt IOP4
Suh, Seung Woo IEP13
Svartman, Celso IOP5
Svehlik, Martin IOP4

Tang, Hao-Che IOP8
Tate, Rebecca IEP7
Theologis, Tim IOP28
Turcot, Katia IEP6

Wang, Matthew N. IOP26
Wang, Ting-Ming IEP4 IOP12
Wang, Stephen IOP21
Wang, Dahui IOP24
Willis, Baxter IOP21
Wu, Kuan-Wen IEP4 IOP12

Yamada, Helder IOP5 IEP5
Yang, Wen-E IOP8 IOP10
Yang, Jae Hyuk IEP13
Yoo, Won Joon IEP11

Open Access

This article is distributed under the terms of the Creative Commons Attribution License which permits any use, distribution, and reproduction in any medium, provided the original author(s) and the source are credited.

